# Supplementary material for: What do blind people “see” with retinal prostheses? Observations and qualitative reports of epiretinal implant users
Source: PLoS One. 2021 Feb 10;16(2):e0229189. doi: 10.1371/journal.pone.0229189 (PMC7875418; doi:10.1371/journal.pone.0229189)
Supplement: Attachment — Submitted filename: Reviewer comments.docx [file pone.0229189.s001.docx]

What do blind people “see” with retinal prostheses? Observations and qualitative reports of epiretinal implant users

This paper discusses the lived experience of epiretinal prosthesis vision recipients across implantation, training, and daily use. The authors argue that a qualitative report of the experience of epiretinal prostheses is a necessary component of assessment and iterative development of epiretinal prostheses. This argument is convincing, however, more could be done to address how: qualitative reports are conducted in clinical trial settings, so that useful comparisons might be made; how qualitative reports of the experience of artificial vision compares to the quantitative measures currently used to assess acuity; and what, if anything, might be changed to improve the qualitative experience of receiving epiretinal artificial vision.

- The paper relies on a form of analysis called Interpretative Phenomenological Analysis. It seems unclear how this analysis derives conclusions from the data. Engineers looking to interpret this analysis in order to make iterative improvements will need to understand the broad strokes of how it operates. A briefly expanded discussion of the data analysis process could greatly assist in the interpretation of results – most especially so for those readers outside the phenomenological ethnography field.
- While it is true that few data are available on the qualitative perception of artificial vision to date, it is not true that there is none. The paper might make brief reference to existing work and note continuity or disparity between experiences. One that jumps to mind is Jens Naumann’s book on Dobelle’s cortical implants. (<https://www.amazon.com/Search-Paradise-Patients-Artificial-Experiment/dp/1479709204>)
- The paper makes no mention of the quantitative results of various psychophysics testing the patients underwent. It seems a missed opportunity to tie qualitative description to quantitative result – if possible, the paper should make every effort to include quantitative results as well.

In general, I would have liked to see a section on recommendations for future design. The interpretation of these results as is is left largely to the engineers who will not understand the analysis. Greater clarity in the underlying data, how analysis was conducted, and how results should be interpreted would significantly improve this paper.

- The paper claims sixteen people were followed, nine of which appear in the paper. A brief note as to why seven people were rejected might improve clarity in the data collection and analysis.
- The authors contradict themselves on data availability, first claiming that all data is fully available without restriction, then claiming patient confidentiality. While it is appropriate for the authors to restrict data availability on this basis, the authors might de-identify data where possible, thereby allowing some data to be shared.
- The figures presented could do more to illustrate the main findings of the paper. Perhaps cartoon drawings of cognitive processes, such as building a scene from a lexicon of flashes, might be more helpful.
- While well-written and presented, there remain a few typos throughout. Lines 571 and 893 are two examples that caught my eye.
